# Preparation of Polyvinylidene Fluoride–Gold Nanoparticles Electrospinning Nanofiber Membranes

**DOI:** 10.3390/bioengineering9040130

**Published:** 2022-03-24

**Authors:** Xuemei Ge, Shang Wu, Wen Shen, Lijuan Chen, Yan Zheng, Fen Ao, Yuanlan Ning, Yueyang Mao, Zhong Chen

**Affiliations:** 1 Department of Food Science and Technology, College of Light Industry Science and Engineering, Nanjing Forestry University, Nanjing 210037, China; gexuemei@njfu.edu.cn (X.G.); 13813972326@163.com (L.C.); 2 School of Food and Bioengineering, Shaanxi University of Science and Technology, Xi’an 710021, China; 210411040@sust.edu.cn (S.W.); zhengyan@sust.edu.cn (Y.Z.); 18309292650@163.com (F.A.); 1904082@sust.edu.cn (Y.N.); 210412103@sust.edu.cn (Y.M.); 3 College of Biological and Pharmaceutical Engineering, Xinyang Agricultural and Forestry University, Xinyang 464000, China; syzsh@antlorchem.com

**Keywords:** electrospinning, polyvinylidene fluoride, gold nanoparticles, nerve regeneration

## Abstract

In this work, gold nanoparticles (AuNPs) and curcumin drug were incorporated in polyvinylidene fluoride (PVDF) nanofibers by electrospinning as a novel tissue engineering scaffold in nerve regeneration. The influence of AuNPs on the morphology, crystallinity, and drug release behavior of nanofiber membranes was characterized. A successful composite nanofiber membrane sample was observed by scanning electron microscopy (SEM). The addition of AuNPs showed the improved as well as prolonged cumulative release of the drug. The results indicated that PVDF–AuNPs nanofiber membrane could potentially be applied for nerve regeneration.

## 1. Introduction

Nerve injury is a universal disease that affects millions of people all over the world, especially young adults [1,2,3,4]. It may cause them to suffer from permanent cognitive, motor, or mental disabilities, increasing their social and economic burden, and reducing their quality of life [5,6]. Although the peripheral nervous system of adult mammals has the ability to self-regenerate [7], without therapeutic interference, spontaneous nerve regeneration may lead to neurological insufficiency. Therefore, the study of tissue-engineered nerve grafts is very necessary [8]. Some experimental and preclinical studies have reported the feasibility of using tissue-engineered nerve grafts as substitutes for autologous nerve grafts to bridge peripheral nerve defects [9,10,11,12]. In recent years, nanofiber nerve grafts developed by electrospinning technology have attracted extensive scientific interest because of their good biocompatibility [13,14,15]. Due to the nano size of fibers, they have many advantages such as large specific surface area and high porosity [16]. It has been proven that electrospun gelatin nerve conduits can promote nerve regeneration [17]. The nerve conduits containing PLGA-SF-Cl prepared by electrostatic spinning method showed a superior repair effect in repairing nerve damage in rats [18].

Electrospinning is currently one of the fabrication methods for the direct and continuous preparation of nanofibers. It is widely used in preparing biomaterials, which could be applied in various fields such as tissue regeneration [19,20,21,22], drug carrier [23,24,25], and wound dressing [26,27,28,29]. It is reported that the electrospun materials combined with peptides could improve the performance of the materials for biological application such as tissue engineering. To overcome the drawbacks of the quick release of the peptide, the covalent linking of these peptides to polymer before or after the electrospinning could be used [30]. PVDF is a five-phase positive piezoelectric polymer with five crystal types of α, β, γ, δ, and ε, among which β, γ, and δ are polar structures [31]. The crystal phase structure of electrostatic spinning fiber membrane mainly exists in the form of β crystal, and other crystal phases can be transformed into β crystal phase by various methods [32]. The existence of β crystal phase is the main reason for the significant piezoelectric effect of PVDF. It is reported that PVDF membrane has antibacterial properties due to their excellent performance [33]. The unique electroactive, flexibility, light weight, and long-time stability under high voltage could offer the feasibility to be applied in extensive fields [34]. The electrospun polymer piezoelectric fiber could mimic the physical, biological as well as material properties of the native extracellular matrix. The PVDF and P(VDF-TrFE) electrospun scaffolds could have an effect on the stimulation of bone repair as well as damaged nerve cells due to the production of the electrical charges under the mechanical deformation [35]. Moreover, the PVDF electrospun fiber could provide the feasibility as the instructive scaffolds for neural stem cell survival and differentiation, and thus could potentially be applied in the field of neural repair [36]. AuNPs have good biocompatibility and excellent metal thermal effect [37], playing an important role in the field of medical detection. AuNPs could be incorporated into nanofibers by electrostatic spinning, and composite nanofibers have properties of both polymers and AuNPs. Using the special properties of PVDF to combine AuNPs, it is possible to prepare two-dimensional nanofiber membranes with piezoelectric effects, providing scaffolds to simulate the extracellular matrix microenvironment. Due to the electrothermal effect of AuNPs embedded in the fibers [38], the release of drugs in the fiber membranes can be promoted. Curcumin is a natural bioactive obtained from powder rhizome of Curcumin longa, which has wide applications in the field of medication, and could also exhibit the effects of promoting regeneration and functional recovery of injured peripheral nerves [39]. Combined with the effects of the scaffold or nanotubes in creating a favorable microenvironment for peripheral nerve regeneration, the curcumin incorporated into the membrane could serve as one option for the purpose of improving nerve regeneration and functional recovery [40,41].

In this paper, PVDF was used to prepare PVDF–AuNPs fiber membrane by electrostatic spinning technology, and the morphology of the fiber membrane was observed by scanning electron microscopy to determine the optimal preparation process conditions. The crystallinity and other properties were characterized. The in vitro release of loading model drug curcumin was carried out. The loading of AuNPs could help to improve the cumulative release of the drug. This drug release model has a promising application in tissue embedding materials.

## 2. Materials and Methods

### 2.1. Materials and Instruments

Polyvinylidene fluoride (PVDF), purchased from Tianjin Jiechuang Yongtai New Material Technology Co., Ltd., Tianjin, China; *N*,*N*-Dimethylformamide (DMF) and ethanol, purchased from Tianjin Kemiou Chemical Reagent Co., Ltd., Tianjin, China; acetone, purchased from Li’an Longbohua (Tianjin) Pharmaceutical and Chemical Co., Ltd., Tianjin, China; gold nanoparticles (10 nm, in sodium citrate solution), purchased from Aladdin Reagent (Shanghai) Co., Ltd., Shanghai, China. Curcumin, purchased from Wuhan Tianzhi Biotechnology Co., Ltd., Wuhan, China; potassium dihydrogen phosphate, disodium hydrogen phosphate, potassium chloride and sodium chloride were all purchased from Tianjin Tianli Chemical Reagent Co., Ltd., Tianjin, China. All the other reagents were conventional commercially available preparations.

FM-1012 electrostatic spinning machine (Beijing Fuyouma Technology Co., Ltd., Beijing, China); D/max2200PC X-ray diffraction (Rigaku, Tokyo, Japan); DSP20 type surface wetting angle measuring instrument (Kruss, Hamburg, Germany); Hitachi S-4800 field emission scanning electron microscope (Hitachi, Tokyo, Japan); Octane prime X-ray photoelectron spectrometer (American EDAX Co., Ltd., Mahwah, NJ, USA); UV-5100 UV-visible spectrophotometer (Shanghai Metash Instrument Co., Ltd., Shanghai, China)

### 2.2. Preparation of Fiber Membranes

PVDF powder was dissolved in DMF and acetone mixed solvent at a certain volume ratio (5:5, 6:4 and 8:2) to prepare the spinning solution with a mass fraction of 12%. The AuNPs with the size of 10 nm in sodium citrate solution (>0.75 A520 Units/mL) were added to the PVDF eletrospinning solution. The concentration of AuNPs was 0.60 nM, which was calculated by determining the absorbance at 520 nm and using an extinction coefficient of 1.25 × 10^9^ M^−1^ cm^−1^ [42,43]. The DMF was mixed with 0.1 mL AuNPs solution, which was stirred at room temperature for 3–4 h with magnetic agitator until completely dissolved. Then, the spinning temperature was adjusted to the test parameters (24 °C, 28 °C, 30 °C, 32 °C, 35 °C and 40 °C), and the distance between the spinneret and the collector was adjusted to 15 cm. The spinning voltage was 23 kV, the jet speed of the spinneret was 0.05 mm/s, and the spinning needle was NO.7 flat head needle. Finally, the spinning fluid was transferred to the syringe and connected with the propulsion device to process the fiber fabrication.

### 2.3. Characterizations

#### 2.3.1. SEM

After the nanofiber membranes were prepared, Hitachi S-4800 field emission scanning electron microscope was used to observe the morphology of the fibers. The samples’ surface was sprayed with gold and SEM was applied to scan the PVDF–Au-formed fiber surface morphology image [44].

#### 2.3.2. XRD

XRD was performed to determine the crystallinity of the PVDF–AuNPs fabricated fiber. Cu target was selected for X-ray diffraction test, and the scanning range was 5~50°. Scanning speed: 4.0000 (deg/min); voltage: 40.0 kV; current: 30.0 mA. The nanofiber membranes were placed on the sample pool for X-ray diffraction determination, and the data were recorded. The crystallinity of the PVDF–AuNPs’ fibers was calculated with Jade6 software.

#### 2.3.3. XPS

X-ray photoelectron spectrometer is a precision instrument that can not only qualitatively analyze the composition on the sample surface, but also quantitatively analyze the content of each component [45]. The five elements of C, N, O, F and Au in the nanofiber membranes were analyzed by X-ray photoelectron spectroscopy.

### 2.4. In Vitro Release Behavior of Model Drug Curcumin

Curcumin was selected as the model drug. A total of 2.5 mg curcumin was dissolved in anhydrous ethanol and mixed with PVDF acetone solution. AuNPs solution of different volumes (0.1 mL, 0.2 mL, 0.3 mL and 0.4 mL) was added to the solution of PVDF–AuNPs before electrospinning.

The curcumin standard curve was set up to calculate the concentration of the curcumin. A total of 10.0 mg of curcumin reference substance was carefully weighed, dissolved, and diluted with anhydrous ethanol to 100 mL, and shaken well to obtain a standard solution. The standard solutions of 0.2, 0.4, 0.6, 0.8, 1.0 and 1.2 mL were further diluted to 10 mL with anhydrous ethanol. The absorbance was measured at wavelength of curcumin at 428 nm. Firstly, PBS buffer was prepared, and oxygen was removed with 800 mL deionized water at 25 °C. Potassium dihydrogen phosphate (KH_2_PO_4_) 0.27 g, disodium hydrogen phosphate (Na_2_HPO_4_) 1.42 g, sodium chloride (NaCl) 8 g, and potassium chloride (KCl) 0.2 g were fully stirred and dissolved. Then concentrated hydrochloric acid was added to adjust pH to 7.4, and the constant volume to 1 L. The dissolution degree was determined by small cup method. Dissolution conditions are as follows. Rotating speed: 50 r/min; temperature: 37 ± 0.5 °C; dissolution medium: PBS buffer; each sample contained 2.5 mg curcumin, and 3 mL samples were taken at 0, 10, 40, 70, 100, 160, 220, 280, 340, and 400 min (supplemented with fresh medium at the same temperature and volume at the same time). The absorbance of the filtrate was measured by UV-VIS spectrophotometer. With PBS buffer as blank control, the absorbance was measured at 428 nm, and the corresponding drug concentration was calculated by using the regression equation of the obtained standard curve and the cumulative dissolution was obtained.

## 3. Results and Discussion

### 3.1. Morphology of Fiber Membranes

**Figure 1:**
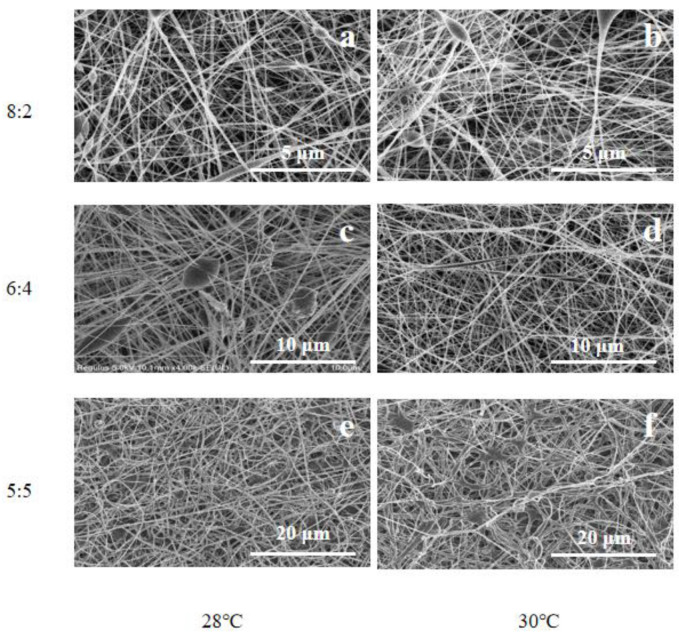
SEM images of fiber membrane at eletrospinning temperature of 28 °C with the DMF to acetone volume ratio of (**a**) 8:2, (**c**) 6:4, (**e**) 5:5; 30 °C with the DMF to acetone volume ratio of (**b**) 8:2, (**d**) 6:4, (**f**) 5:5.

As shown in Figure 1, the pure PVDF fiber membranes obtained under different process conditions are observed. It can be found that different solvent volume ratio and temperature have a great influence on the morphology of PVDF fiber membrane. When the volume ratio of DMF and acetone is 8:2, there are large droplets in the obtained fiber membrane. This is because the acetone content in the spinning solution is low and the volatilization performance is poor; during electrospinning, the viscosity of spinning solution is high and the stretching degree of fiber is not enough, so a more obvious fiber membrane with droplets is formed. When the volume ratio is 6:4, the droplets are significantly reduced, and the fiber diameter distribution is more uniform. This is because from the increase in acetone content, the volatilization performance of the spinning solution is improved, and the viscosity of the spinning solution is reduced, which is conducive to refining and stretching into nanofibers in the electrospinning process. By comparing the effects of temperature on the morphology of the fiber membrane at the same solvent and volume ratio, it is found that the morphology of the fiber membrane is better at 28 °C, the droplets are fewer, and the thickness distribution is uniform.

**Figure 2:**
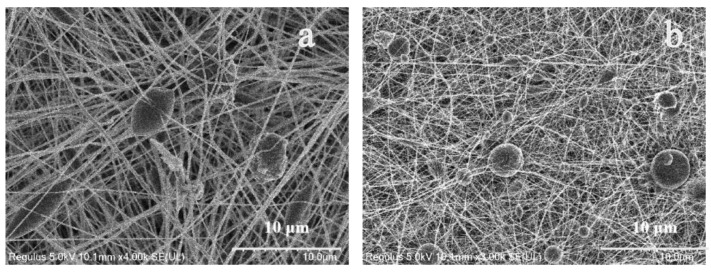
SEM images. (**a**) PVDF electrospun nanofibers under the process condition of 28 °C and 6:4 solvent volume ratio; (**b**) PVDF–AuNPs electrospun nanofibers under the condition of 28 °C and 6:4 solvent volume ratio.

**Figure 3:**
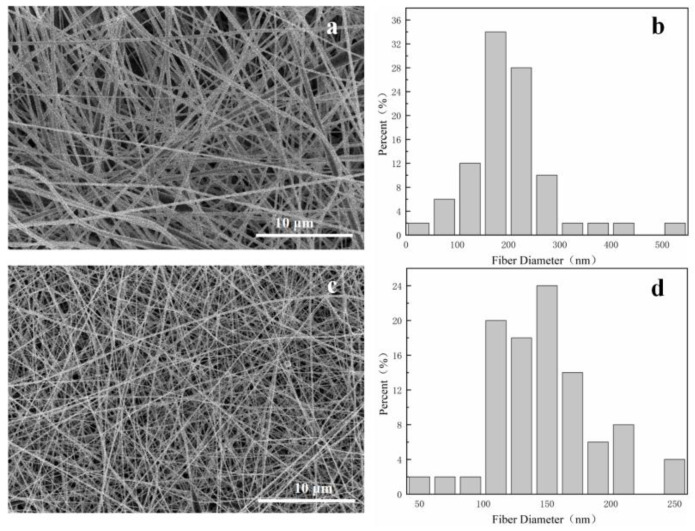
(**a**) SEM images of PVDF electrospun nanofibers under the process condition of 28 °C and 5:5 solvent volume ratio; (**b**) Diameter distribution histograms of (**a**). (**c**) SEM images of PVDF–AuNPs electrospun nanofibers under the process condition of 28 °C and 5:5 solvent volume ratio (**b**). (**d**) Diameter distribution histograms of (**c**).

It can be seen from the above that when the volume ratio of DMF and acetone is 6:4 and the temperature is 28 °C, the morphology of the prepared fiber membrane is good, as shown in Figure 1c. Compared with PVDF nanofiber membrane, the fiber diameter is significantly smaller after gold loading. This can be observed in Figure 2. Figure 3 shows that the morphology of pure PVDF fiber membrane and PVDF–AuNPs fiber membrane under the process condition of 28 °C and 5:5 solvent volume ratio, and it also can be clearly observed that the diameter of the fiber membrane is smaller. The reason for this may be that the added AuNPs produce a thermal effect in the electrospinning process, resulting in the transformation of PVDF molecular segments from crystalline to amorphous, which is conducive to the refinement and stretching of droplets.

As a kind of noble metal, AuNPs also increased the conductivity of the spinning fluid, which carried more electric charges during electrospinning, so that more electric splitting refining processes could take place. The electric field force acting on the surface of the droplet was increased, and its thinning and stretching capacity was enhanced. The diameter of the electrospinning fiber was smaller.

### 3.2. XRD

Through XRD characterization of PVDF–AuNPs fiber membranes, it can be found that the fiber membranes are mainly β crystalline after electric field force polarization and stretching, and the average crystallinity of the obtained fiber membranes is 14.18%. The crystallinity of PVDF powder is about 34%. The change of crystallinity mainly depends on the transformation of the PVDF crystal form. After electrospinning, the crystallinity of the fiber membranes decreases, which proves that the content of β phase in the fiber membranes increases, and the piezoelectric effect increases accordingly. The reason may be that the loading of AuNPs increases the conductivity of the spinning solution used in electrostatic spinning and promotes the stretching of PVDF from α spherulite structure to the microfibrous β crystal structure.

**Figure 4:**
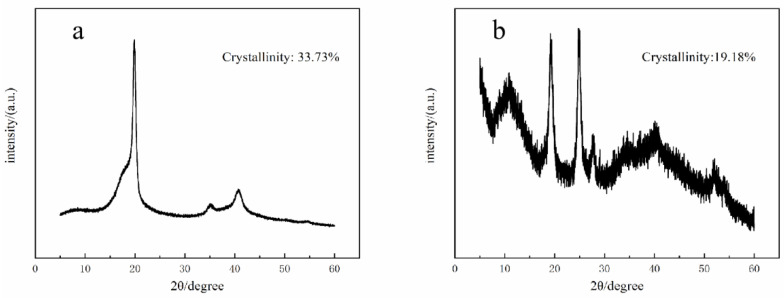
(**a**) XRD image of non-modified PVDF powder (**b**) XRD image of fiber membrane of the PVDF fiber fabricated together with AuNPs.

Figure 4 shows the XRD characterization results of the fiber membranes prepared under the conditions of DMF and acetone at a volume ratio of 6:4 and a temperature of 28 °C. The diffraction peaks of the fiber membrane at 2θ of 10.4°, 19.2° and 27.8° are observed. In the figure, it is obvious that the peak intensity corresponding to the β phase is stronger, which indicates that the crystal shape of gold-loaded PVDF after polarization and stretching by electrostatic spinning is still mainly composed of polar β phase, and the crystallinity of gold-loaded PVDF fiber membrane is low.

### 3.3. XPS

**Figure 5:**
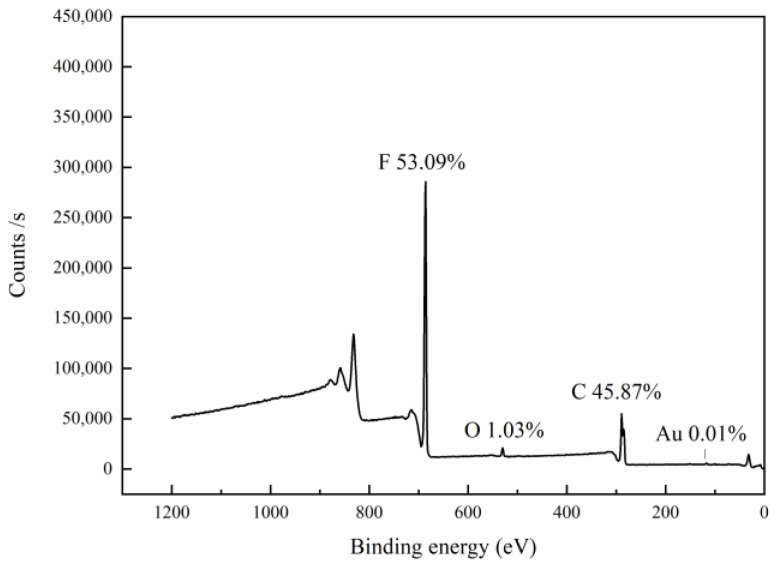
XPS image of fiber membrane of the PVDF fiber fabricated together with AuNPs.

Figure 5 shows the photoelectron spectroscopy of PVDF–AuNPs fiber membrane, which reveals that the constituent elements of the fiber membrane are mainly carbon and fluorine. At the same time, through the fine spectrum of the fiber membrane, it is found that fluorine and carbon mainly exist in the valence states of F1s and C1s without other additional peaks. It shows that the surface of the fiber membrane is not polluted, and it also shows that more AuNPs are embedded in the nanofibers.

### 3.4. In Vitro Release of Model Drugs

**Figure 6:**
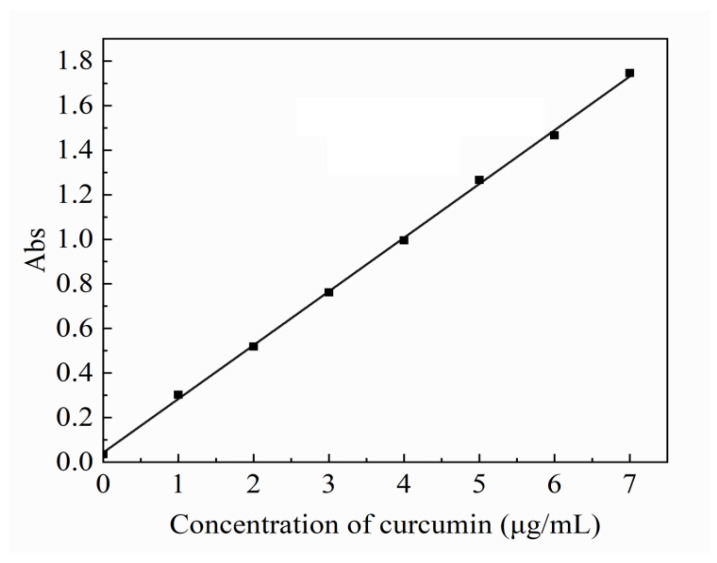
Standard curve of curcumin as the model drug.

The standard curve of curcumin is shown in Figure 6. The standard equation of curcumin release is y = 0.2412x + 0.0425, R^2^ = 0.9996 (x is the concentration of curcumin, y is the absorbance), and the linear relationship is good.

**Figure 7:**
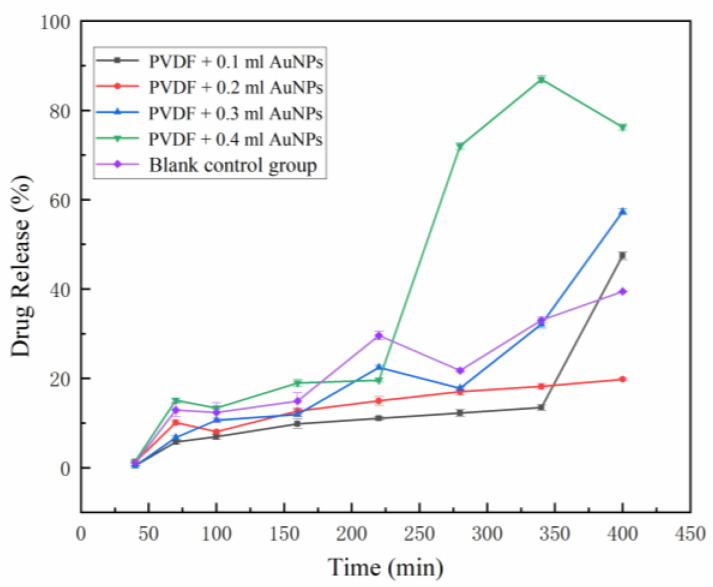
Cumulative release rate curve of drug-loaded fiber membrane in different groups: PVDF + 0.1 mL AuNPs, PVDF + 0.2 mL AuNPs, PVDF + 0.3 mL AuNPs, PVDF + 0.4 mL AuNPs, blank control group. The concentration of the AuNPs was 0.60 nM.

Figure 7 shows drug release from drug-loading fiber membrane. In the cumulative release curve, fiber membrane loaded with smaller amounts of AuNPs at the volumes of 0.1 mL, 0.2 mL, 0.3 mL, and 0.4 mL at the first 5 h cumulatively release less than the blank control group. This is because the amount of AuNPs loaded in the fiber membrane at the initial stage of release is not enough to produce the thermal effect promoting drug release, and the gold nanoparticles mixed in the spinning solution have a chelation or adsorption effect on curcumin, leading to the release variation. However, with the lapse of dissolution time, the release rate of the blank control group showed a slowing trend in the subsequent release of 5–9 h, while the release rate of gold-loaded fiber membrane increased, indicating that the thermal effect of AuNPs in the fiber gradually accumulated during the solution agitation process and reduced the crystallization degree of PVDF nearby. The drug release curve of PVDF membrane with low AuNPs content showed a slow and controlled release effect with a time-dependent effect.

## 4. Conclusions

The optimized conditions for electrospinning PVDF–AuNPs membranes were as follows: DMF and acetone volume ratio 6:4, spinning temperature 28 °C. The fiber membrane could have good morphology and no obvious defects can be obtained. The fiber membrane has low crystallinity, large β phase crystal area, and good AuNPs embedding effect. In PVDF fiber, AuNPs had the double cumulative effect of time and content, and a small amount of AuNPs was not enough to produce the thermal effect to promote the release of curcumin in a short time. When the addition of AuNPs increased, the electrothermal effect could obviously improve the release of curcumin.

## Data Availability

Not applicable.

## References

[1] Daly W., Yao L., Zeugolis D., Windebank A., Pandit A. (2012). A biomaterials approach to peripheral nerve regeneration: Bridging the peripheral nerve gap and enhancing functional recovery. J. R. Soc. Interface.

[2] Li C., Liu S.-Y., Pi W., Zhang P.-X. (2021). Cortical plasticity and nerve regeneration after peripheral nerve injury. Neural Regen. Res..

[3] Anwar H., Rasul A., Iqbal J., Ahmad N., Imran A., Malik S.A., Ijaz F., Akram R., Maqbool J., Sajid F. (2021). Dietary biomolecules as promising regenerative agents for peripheral nerve injury: An emerging nutraceutical-based therapeutic approach. J. Food Biochem..

[4] Srihagulang C., Vongsfak J., Vaniyapong T., Chattipakorn N., Chattipakorn S.C. (2022). Potential roles of vagus nerve stimulation on traumatic brain injury: Evidence from in vivo and clinical studies. Exp. Neurol..

[5] Wijntjes J., Borchert A., van Alfen N. (2021). Nerve ultrasound in traumatic and iatrogenic peripheral nerve injury. Diagnostics.

[6] Zhang R.-C., Du W.-Q., Zhang J.-Y., Yu S.-X., Lu F.-Z., Ding H.-M., Cheng Y.-B., Ren C., Geng D.-Q. (2021). Mesenchymal stem cell treatment for peripheral nerve injury: A narrative review. Neural Regen. Res..

[7] Jones S., Eisenberg H.M., Jia X. (2016). Advances and future applications of augmented peripheral nerve regeneration. Int. J. Mol. Sci..

[8] Wang B., Lu C.-F., Liu Z.-Y., Han S., Wei P., Zhang D.-Y., Kou Y.-H., Jiang B.-G. (2022). Chitin scaffold combined with autologous small nerve repairs sciatic nerve defects. Neural Regen. Res..

[9] Friedrich R.P., Cicha I., Alexiou C. (2021). Iron oxide nanoparticles in regenerative medicine and tissue engineering. Nanomaterials.

[10] Ghaderinejad P., Najmoddin N., Bagher Z., Saeed M., Karimi S., Simorgh S., Pezeshki-Modaress M. (2021). An injectable anisotropic alginate hydrogel containing oriented fibers for nerve tissue engineering. Chem. Eng. J..

[11] Xue W., Shi W., Kong Y., Kuss M., Duan B. (2021). Anisotropic scaffolds for peripheral nerve and spinal cord regeneration. Bioact. Mater..

[12] Li G., Zhang B., Sun J.-H., Shi L.-Y., Huang M.-Y., Huang L.-J., Lin Z.-J., Lin Q.-Y., Lai B.-Q., Ma Y.-H. (2021). An nt-3-releasing bioscaffold supports the formation of trkc-modified neural stem cell-derived neural network tissue with efficacy in repairing spinal cord injury. Bioact. Mater..

[13] Miller R.J., Chan C.Y., Rastogi A., Grant A.M., White C.M., Bette N., Schaub N.J., Corey J.M. (2018). Combining electrospun nanofibers with cell-encapsulating hydrogel fibers for neural tissue engineering. J. Biomater. Sci. Polym. Ed..

[14] Zhang W., Weng T., Li Q., Jin R., You C., Wu P., Shao J., Xia S., Yang M., Han C. (2021). Applications of poly(caprolactone)-based nanofibre electrospun scaffolds in tissue engineering and regenerative medicine. Curr. Stem Cell Res. Ther..

[15] Zhang M., Li C., Zhou L.-P., Pi W., Zhang P.-X. (2021). Polymer scaffolds for biomedical applications in peripheral nerve reconstruction. Molecules.

[16] Kim J.I., Kim C.S., Park C.H., Chun H.J., Park C.H., Kwon I.K., Khang G. (2018). Harnessing nanotopography of electrospun nanofibrous nerve guide conduits (ngcs) for neural tissue engineering. Cutting-Edge Enabling Technologies for Regenerative Medicine.

[17] Cirillo V., Clements B.A., Guarino V., Bushman J., Kohn J., Ambrosio L. (2014). A comparison of the performance of mono- and bi-component electrospun conduits in a rat sciatic model. Biomaterials.

[18] Zhang M., Lin W., Li S., Shi X.-Y., Liu Y., Guo Q., Huang Z., Li L., Wang G.-L. (2016). Application and effectiveness evaluation of electrostatic spinning plga-silk fibroin-collagen nerve conduits for peripheral nerve regeneration. J. Nanosci. Nanotechnol..

[19] Xie X., Chen Y., Wang X., Xu X., Shen Y., Khan A.U.R., Aldalbahi A., Fetz A.E., Bowlin G.L., El-Newehy M. (2020). Electrospinning nanofiber scaffolds for soft and hard tissue regeneration. J. Mater. Sci. Technol..

[20] Chen W., Xu Y., Li Y., Jia L., Mo X., Jiang G., Zhou G. (2020). 3d printing electrospinning fiber-reinforced decellularized extracellular matrix for cartilage regeneration. Chem. Eng. J..

[21] Kumar T.S.S., Chakrapani V.Y., Chun H.J., Park C.H., Kwon I.K., Khang G. (2018). Electrospun 3d scaffolds for tissue regeneration. Cutting-Edge Enabling Technologies for Regenerative Medicine.

[22] Xu X., Ren S., Li L., Zhou Y., Peng W., Xu Y. (2021). Biodegradable engineered fiber scaffolds fabricated by electrospinning for periodontal tissue regeneration. J. Biomater. Appl..

[23] Zheng X., Kang S., Wang K., Yang Y., Yu D.-G., Wan F., Williams G.R., Bligh S.-W.A. (2021). Combination of structure-performance and shape-performance relationships for better biphasic release in electrospun janus fibers. Int. J. Pharm..

[24] Topuz F., Uyar T. (2019). Electrospinning of cyclodextrin functional nanofibers for drug delivery applications. Pharmaceutics.

[25] Farokhi M., Mottaghitalab F., Reis R.L., Ramakrishna S., Kundu S.C. (2020). Functionalized silk fibroin nanofibers as drug carriers: Advantages and challenges. J. Control Release.

[26] Chen K., Pan H., Ji D., Li Y., Duan H., Pan W. (2021). Curcumin-loaded sandwich-like nanofibrous membrane prepared by electrospinning technology as wound dressing for accelerate wound healing. Mater. Sci. Eng. C-Mater. Biol. Appl..

[27] Zou P., Lee W.-H., Gao Z., Qin D., Wang Y., Liu J., Sun T., Gao Y. (2020). Wound dressing from polyvinyl alcohol/chitosan electrospun fiber membrane loaded with oh-cath30 nanoparticles. Carbohydr. Polym..

[28] Yang J., Wang K., Yu D.-G., Yang Y., Bligh S.W.A., Williams G.R. (2020). Electrospun janus nanofibers loaded with a drug and inorganic nanoparticles as an effective antibacterial wound dressing. Mater. Sci. Eng. C-Mater. Biol. Appl..

[29] Jeckson T.A., Neo Y.P., Sisinthy S.P., Gorain B. (2021). Delivery of therapeutics from layer-by-layer electrospun nanofiber matrix for wound healing: An update. J. Pharm. Sci..

[30] Bucci R., Vaghi F., Erba E., Romanelli A., Gelmi M.L., Clerici F. (2021). Peptide grafting strategies before and after electrospinning of nanofibers. Acta Biomater..

[31] Gade H., Bokka S., Chase G.G. (2021). Polarization treatments of electrospun pvdf fiber mats. Polymer.

[32] Wang A., Shao M., Yang F., Shao C., Chen C. (2021). Preparation and properties of antibacterial pvdf composite thin films. Eur. Polym. J..

[33] Singh R.K., Lye S.W., Miao J. (2021). Holistic investigation of the electrospinning parameters for high percentage of beta- phase in pvdf nanofibers. Polymer.

[34] Ahn Y., Lim J.Y., Hong S.M., Lee J., Ha J., Choi H.J., Seo Y. (2013). Enhanced piezoelectric properties of electrospun poly(vinylidene fluoride)/multiwalled carbon nanotube composites due to high beta-phase formation in poly(vinylidene fluoride). J. Phys. Chem. C.

[35] Li Y., Liao C., Tjong S.C. (2019). Electrospun polyvinylidene fluoride-based fibrous scaffolds with piezoelectric characteristics for bone and neural tissue engineering. Nanomaterials.

[36] Lins L.C., Wianny F., Livi S., Dehay C., Duchet-Rumeau J., Gerard J.-F. (2017). Effect of polyvinylidene fluoride electrospun fiber orientation on neural stem cell differentiation. J. Biomed. Mater. Res. Part B-Appl. Biomater..

[37] Shiue A., Chen J.-H., Chang C.-Y., Chang S.-M., Hwa K.-Y., Chin K.-Y., Leggett G. (2020). Synthesis and cytotoxic analysis of thiolated xylose derivatives decorated on gold nanoparticles. Biotechnol. Rep..

[38] Jaswal R., Shrestha S., Shrestha B.K., Kumar D., Park C.H., Kim C.S. (2020). Nanographene enfolded aunps sophisticatedly synchronized polycaprolactone based electrospun nanofibre scaffold for peripheral nerve regeneration. Mater. Sci. Eng. C-Mater. Biol. Appl..

[39] Zhao Z., Li X., Li Q. (2017). Curcumin accelerates the repair of sciatic nerve injury in rats through reducing schwann cells apoptosis and promoting myelinization. Biomed. Pharmacother..

[40] Moattari M., Moattari F., Kouchesfahani H.M., Kaka G., Sadraie S.H., Naghdi M., Mansouri K. (2018). Curcumin and biodegradable membrane promote nerve regeneration and functional recovery after sciatic nerve transection in adult rats. Ann. Plast. Surg..

[41] Jahromi H.K., Farzin A., Hasanzadeh E., Barough S.E., Mahmoodi N., Najafabadi M.R.H., Farahani M.S., Mansoori K., Shirian S., Ai J. (2020). Enhanced sciatic nerve regeneration by poly-l-lactic acid/multi-wall carbon nanotube neural guidance conduit containing schwann cells and curcumin encapsulated chitosan nanoparticles in rat. Mater. Sci. Eng. C-Mater. Biol. Appl..

[42] Aslan K., Luhrs C.C., Perez-Luna V.H. (2004). Controlled and reversible aggregation of biotinylated gold nanoparticles with streptavidin. J. Phys. Chem. B.

[43] Haiss W., Thanh N.T.K., Aveyard J., Fernig D.G. (2007). Determination of size and concentration of gold nanoparticles from uv-vis spectra. Anal. Chem..

[44] Vazquez-Vazquez E.F., Rojas-Chavez H., Hernandez-Rodriguez Y.M., Morales-Bautista J., Cigarroa-Mayorga O.E. (2021). The role of aunps on the photocatalytic degradation enhancement in moo3-based heterostructures. Mater. Lett..

[45] Abolhasani M.M., Azimi S., Fashandi H. (2015). Enhanced ferroelectric properties of electrospun poly(vinylidene fluoride) nanofibers by adjusting processing parameters. RSC Adv..

